# Charge-Shift Bonding Propensity in Halogen-Bonded BXY (B Is a Small Lewis Base H_2_O or NH_3_; X and Y Are Halogen Atoms) Complexes: An NBO/NRT/AIM Investigation

**DOI:** 10.3390/molecules28176212

**Published:** 2023-08-24

**Authors:** Junjie Song, Mengyang Wang, Xiaocheng Xu, Qinghao Shao, Ying Zhao, Guiqiu Zhang, Nan Sun

**Affiliations:** 1 Key Laboratory of Molecular and Nano Probes, Ministry of Education, College of Chemistry, Chemical Engineering and Materials Science, Shandong Normal University, Jinan 250014, China; 2 Key Laboratory of Organic Optoelectronics and Molecular Engineering of the Ministry of Education, Department of Chemistry, Tsinghua University, Beijing 100084, China

**Keywords:** CS bonding propensity, ω-bonding, halogen bonding, NBO/NRT, AIM

## Abstract

Charge-shift (CS) bonding is a new bonding paradigm in the field of chemical bonds. Our recent study has revealed that certain Cu/Ag/Au-bonds display both CS bonding and ω-bonding characters. In this investigation, we extend our study to halogen bonding. Our focus is on scrutinizing the CS bonding in halogen-bonded BXY (B is a small Lewis base H_2_O or NH_3_; X and Y are halogen atoms) complexes by using natural bond orbital (NBO) analysis, natural resonance theory (NRT), and atoms in molecules (AIM) methods. The primary objective is to establish a connection between halogen bonding (B–X) in BXY and CS bonding in free XY (di-halogens). The calculations indicate that the studied BXY can be classified into two types. One type with a weak halogen bond shows closed-shell interaction. The other type with a stronger B–X interaction exhibits both CS bonding and ω-bonding characters (as seen in NH_3_ClF, NH_3_BrF, and NH_3_IF). Another interesting finding is a novel propensity that the CS bonding in free XY tends to carry over the halogen bonding in BXY, and the same propensity is found in Cu/Ag/Au ω-bonded species. The present study may offer an approach to probe CS bonding in many more 3c/4e ω-bonded molecules.

## 1. Introduction

The chemical bond serves as the foundation of chemistry. It provides a framework for the classification and prediction of new compounds. In the field of chemical bonds, charge-shift (CS) bonding has emerged as a new bonding paradigm. A task, therefore, lies ahead to find the CS bonding in a wider variety of molecules.

Alongside traditional covalent and ionic bonds, the CS bond, as a new kind of electron-pair bond, was first introduced by Shaik and Hiberty et al. [1] in 1992. Unlike covalent or ionic bonds, CS bond energy arises partly from significant resonance energy. A classic example is the F–F bond in F_2_. Within the framework of valence bond (VB) theory, this bond can be described as a hybrid of two Lewis structures: F–F ↔ F^+^ F^−^, whose resonance energy is 62.2 kcal/mol and relative resonance energy (RRE) is up to 183.9% (total bond energy of 33.8 kcal/mol) [2]. Moreover, CS bonding can also be characterized within the atoms in molecules (AIM) [3] framework by using the combination of a significant total electron density *ρ*(r) along with a positive (or small) Laplacian ∇^2^*ρ*(r) at the bond critical points (BCPs) [4]. Notably, it has been reported that the characterization of CS bonding remains consistent across different methods [5,6].

In the frameworks of VB and AIM theories, Shaik and coworkers have demonstrated that certain single two-center bonds such as Au–Au [7], Au–F [8], Cl–F [9], and Cl–Cl [4] in diatomic molecules, as well as metal–metal bonds [10,11] in traditional metal complexes, exhibit significant CS bonding characters. Apart from these species, CS bonding is also notably prominent in hypervalent molecules, particularly in some electron-rich three-center/four-electron (3c/4e) species, such as the XeF_2_ [12]. Similar results are observed for other typical hypervalent molecules like SF_4_, PF_5_, and ClF_3_ [13]. Of course, CS bonding can also be derived through other approaches. For instance, natural bond orbital (NBO) and natural resonance theory (NRT) analyses, along with experimental vibrational frequency shifts of the studied bond, revealed that the H–Xe bond in the HXeF molecule belongs to the CS bonding family [14]. Another example is that the Cu/Ag/Au bonds (also known as coinage-metal bonds) [15] in BMY (B = H_2_O, H_2_S, NH_3_, and PH_3_; M = Cu, Ag, and Au; and Y = F, Cl, Br, and I) complexes have been established to show the characters of both CS bonding and ω-bonding in our recent work [16] by using the NBO/NRT/AIM methods. Additional examples of CS bonding could be found in recent publications [17,18,19,20]. Here, it is necessary to introduce the ω-bonding model because the studied halogen bonding, as shown in the following, exhibits both CS bonding and ω-bonding characters. The ω-bonding is another quantum chemically derived model. Weinhold et al. [21] were the first to propose this model and to rationalize the bonding in several 3c/4e hypervalent molecules by considering the strong resonance of two natural Lewis structures. Taking F_3_^−^ as one example, its bonding could be described as a resonance hybrid of two natural Lewis structures, F–F F^−^ ↔ F^−^ F–F, originating in hyperconjugative interactions. The distinctive characteristics of ω-bonding are equivalent or close weights of two resonance structures. The great merit of the ω-bonding model is to provide a resonance bonding picture. It can allow a straightforward explanation and qualitative prediction of stability for 3c/4e hypervalent molecules. It is noteworthy that both CS bonding and ω-bonding models can provide a fundamental description of the resonance bonding for 3c/4e hypervalent molecules.

Overall, the field of this new bond is actively expanding, with continuous advancements in probing techniques and the continual addition of new molecules to the CS family. Given that halogen-bonded complex BXY (B is a small Lewis base; X and Y are halogen atoms) and Cu/Ag/Au-bonded complex BMY have resemblances in geometrical and electronic structures, we raise two questions: (1) Does the halogen bonding (B–X) in BXY belong to the CS bonding family? (2) If the answer is “yes”, is there a connection of CS bonding nature between the halogen bond in BXY and the X–Y bond in free di-halogens (XY)? To address these questions, this study chooses halogen-bonded BXY (B is H_2_O or NH_3_) complexes as the study systems. Some of them have been characterized in spectroscopic experiments [22,23,24,25,26]. Then, the bonding mechanism, nature, and propensity are analyzed by using the NBO/NRT/AIM methods based on the optimized BXY structures.

Of course, the present work is restricted to halogen bonding. Nevertheless, as we shall discuss in the following, the conclusions are related to both halogen bonding and Cu/Ag/Au bonding. Our studies show that CS bonding occurs in both stronger halogen and Cu/Ag/Au bonded complexes. Note that this result has a profound impact on how we understand such CS bonding that was previously thought to be “non-covalent interaction”. As we know, the term halogen bond is introduced for describing the interaction involving halogens as acceptors of electron density. It is often loosely classified as non-covalent interactions. However, Hobza et al. stated the following: “This name (non-covalent interaction) is not perfect, as some strong interactions, for example, dative and charge-transfer interactions, lie somewhere between the noncovalent and covalent realms” [27]. Also note that the CS bond is a new type of electron-pair bond. It is different from the classical covalent bond in its bonding mechanism and bond strength. Our examined stronger halogen bonds and Cu/Ag/Au bonds are classified as CS covalent interaction. Therefore, they are neither classical covalent bonds nor perfect non-covalent interactions.

## 2. Results and Discussion

### 2.1. Geometries and Dissociation Energies

**Figure 1:**
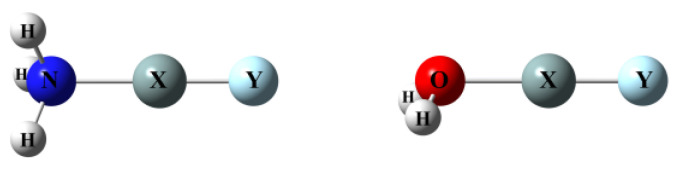
Geometries of BXY, where B is H_2_O or NH_3_; X and Y are halogen atoms.

The models of the optimized geometry are presented in Figure 1 for BXY complexes, which agree with the experimental characterizations that the series of NH_3_XY have *C*_3v_ symmetry, while each of the H_2_OXY species has *C*_s_ symmetry, with N/O, X, and Y atoms lying on an axis [23,24,25,26]. The calculated equilibrium B–X and X–Y bond lengths (*r*) at the MP2/aug-cc-pVTZ (-PP) computational level are listed in Table 1. Here, the cases of X being Au also are introduced to compare the halogen bonds with the coinage-metal bonds. It can be seen that the lengths of the B–X bond containing identical Lewis base B and atom X are in the order of F < Cl < Br < I. Taking the series of NH_3_IY for one illustrative instance, the N–I bond lengths are 2.443, 2.540, 2.583, and 2.672 Å for NH_3_IF, NH_3_ICl, NH_3_IBr, and NH_3_II, respectively. This indicates that the interaction between B and X will weaken with the order of F, Cl, Br, and I. In addition, the equilibrium bond lengths between the halogen atoms in BXY complexes are larger than those in the free di-halogens, especially when the Lewis base NH_3_ is present. For example, the *r*_I–F_ is 1.920, 1.941, and 1.976 Å for IF, H_2_OIF, and NH_3_IF, respectively. Table S1 in the Supporting Information (SI) collects the calculated vibrational frequencies (*ν*) of B–X and X–Y bonds at the MP2 level, which gives the results corresponding to the changing trends of the bond lengths. The intermolecular vibrational frequency *ν*_B–X_ of the B–X bond in BXY varies and becomes significant with the order of F, Cl, Br, and I. For example, the *ν*_N–I_ is 272, 218, 202, and 173 cm^−1^ for NH_3_IF, NH_3_ICl, NH_3_IBr, and NH_3_II, respectively. The *ν*_X–Y_ are found red shifts in complexes, when they are compared in XY and BXY, especially in NH_3_XY species. For instance, the *ν*_I–F_ is 634, 604, and 558 cm^−1^ for IF, H_2_OIF, and NH_3_IF, respectively. Figure S1 in the SI plots the correlations between *r*_B–X_ and *ν*_B–X_ for NH_3_IY and NH_3_AuY series. As expected, there is a clear negative correlation between *r*_B–X_ and *ν*_B–X_, according to the familiar Pearson χ^2^ coefficients [28], whose values are 0.977 and 0.999 for *r*_N–I_-*ν*_N–I_ and *r*_N–Au_-*ν*_N–Au_ correlations, respectively.

**Figure 2:**
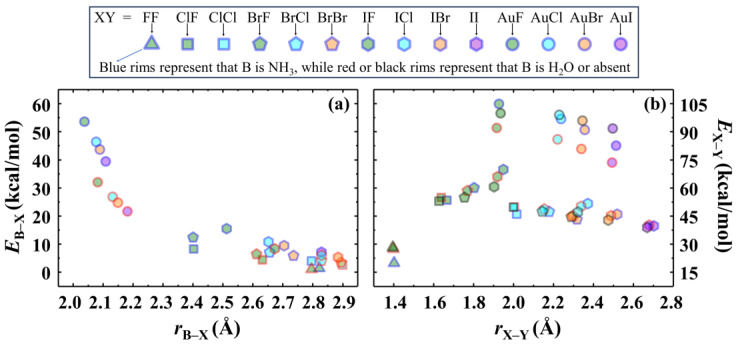
Calculated bond length (*r*) and dissociation energy (*E*) of B–X (**a**) and X–Y (**b**) bonds in all studied species at the CCSD/def2-TZVPPD computational level. The blue, red, and black rims represent that the Lewis base B is NH_3_, H_2_O, and absent (free XY), respectively. The symbols triangle, square, pentagon, hexagon, and circle represent that the atom X is F, Cl, Br, I, and Au, respectively. The green, cyan, orange, and purple centers represent that the atom Y is F, Cl, Br, and I, respectively.

The calculated dissociation energies (*E*) of B–X and X–Y bonds for all studied species at the MP2 level are summarized in Table S2 in the SI, showing the energies for both Lewis base B removal (BXY → B + XY, *E*_B–X_ = *E*_(B)_ + *E*_(XY)_ − *E*_(BXY)_) and halogen atom Y removal (BXY → BX + Y, *E*_X–Y_ = *E*_(BX)_ + *E*_(Y)_ − *E*_(BXY)_) of BXY complexes, as well as the neutral atomic dissociation (XY → X + Y, *E*_X–Y_ = *E*_(X)_ + *E*_(Y)_ − *E*_(XY)_) of free XY molecules. Note that the MP2/aug-cc-pVTZ (-PP) computational level for homolytic bond cleavage enthalpies of the molecules we tested is quite different from the higher CCSD (T) level. The following Computational Details section involves the comparisons of many computational levels. We found that the calculation results at the CCSD level are comparable to those at the CCSD (T) level, and it can significantly reduce the calculation cost. Hence, the dissociation energies we will discuss below are all calculated at the CCSD level. Nevertheless, all subsequent analyses in this paper are still based on the optimized molecular structures at the MP2 level because their performance in calculating geometry is more consistent with the experimental results. The calculated equilibrium bond lengths, vibrational frequencies, and dissociation energies at the CCSD/def2-TZVPPD computational level are presented in Tables S3–S5, respectively. Figure 2a,b show the calculated equilibrium lengths and dissociation energies of B–X and X–Y bonds, respectively. As we can see, the dissociation energies of all halogen bonds, except for those in a few BXY complexes, are less than 10 kcal/mol. It is worth noting that the dissociation energy of the studied halogen bonds does not have a good linear relationship with the bond length. For example, the *E*_N–I_ values are 15.51, 10.82, 9.46, and 7.15 kcal/mol, while the *r*_N–I_ values are 2.512, 2.652, 2.704, and 2.829 Å for NH_3_IF, NH_3_ICl, NH_3_IBr, and NH_3_II, respectively (χ^2^ = 0.973). What is more, the dissociation energy of the bond between two halogen atoms even increases with increasing bond length. For example, the I–F bond lengths of IF, H_2_OIF, and NH_3_IF increase gradually, and the corresponding *E*_I–F_ values are 60.56, 65.93, and 69.75 kcal/mol, respectively. These unexpected phenomena suggest that the bond strength of some BXY complexes may require the consideration of other contributions, such as the “charge-shift resonance energy” resulting from covalent–ionic mixing.

### 2.2. 3c/4e ω-Bonding in a Halogen-Bonded Complex

The NBO/NRT analysis is the most frequently used tool for analyzing 3c/4e ω-bonding [21]. Based on NBO analysis, the best single natural Lewis structure (NLS) can be immediately confirmed for one studied molecule, and the donor–acceptor orbital interactions in it can be quantitatively estimated [43]. The NRT algorithms are built on NBO-based donor–acceptor concepts to provide the numerical resonance weightings of contributing resonance structures [44,45,46]. The coinage-metal bonded BAuY series have been identified as the 3c/4e ω-bonded molecules, according to the proposal by Weinhold et al. [21] such as the linear B–Au–Y geometry, strong n_B_→σ*_Au–Y_ and n_Y_→σ*_B–Au_ donor–acceptor interactions, high populations of σ*_B–Au_ and σ*_Au–Y_ antibonds, and relatively close resonance weightings. Therefore, we employ the NBO/NRT method to investigate if the studied halogen-bonded BXY meet the criteria of 3c/4e ω-bonding.

**Figure 3:**
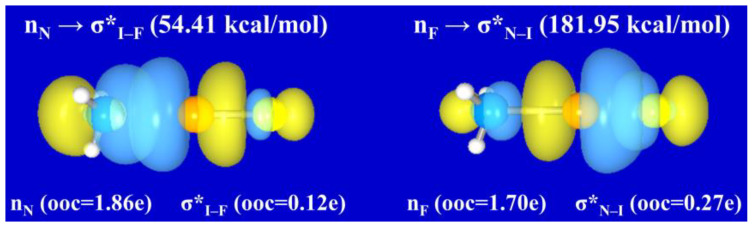
Three-dimensional surface views of the interactions n_N_ → σ*_I–F_ and n_F_ → σ*_N–I_ in NH_3_IF.

Table S6 in the SI summarizes the calculated second-order perturbation stabilization energies (Δ*E*^(2)^_D→A_), estimating the n_B_→σ*_X–Y_ and n_Y_→σ*_B–X_ donor–acceptor interactions in BXY complexes. For n_B_→σ*_X–Y_ interaction, the donor is the lone pair orbital (n_B_) of Lewis base B, and the acceptor is the antibonding orbital (σ*_X–Y_) of X–Y moiety in B: X–Y structure. In the complementary resonance structure B–X^+^:Y^−^, the n_Y_→σ*_B–X_ interaction involves the lone pair n_Y_ and the antibonding orbital σ*_B–X_. The donor–acceptor interaction is usually accompanied by a transfer of electron density. Table S7 in the SI presents the calculated orbital occupancies (e) of the lone pair orbital and the antibonding orbital arising from the n_B_→σ*_X–Y_ or n_Y_→σ*_B–X_ interaction. On the basis of its significantly lower Δ*E*^(2)^_D→A_ (Table S6) and lower orbital occupancy of σ*_X–Y_ (Table S7), it can be predicted that the resonance structure B: X–Y is absolutely dominant for most halogen-bonded complexes. Nevertheless, there are still a few complexes whose resonance structure B–X^+^:Y^−^ can not be ignored, such as NH_3_ClF, NH_3_BrF, and NH_3_IF. Taking NH_3_IF species as an example, the 3D surface views and the orbital overlap contour diagrams of its n_N_→σ*_I–F_ and n_F_→σ*_N–I_ donor-acceptor interactions are depicted in Figure 3 and Figure S2, respectively. The second-order perturbation stabilization energy for n_N_→σ*_I–F_ is 54.41 kcal/mol, and that for n_F_→σ*_N–I_ is 181.95 kcal/mol. Furthermore, the n_N_→σ*_I–F_ interaction brings an electron transfer of about 0.12e from the lone pair orbital n_N_ to the antibonding orbital σ*_I–F_, while the n_F_→σ*_N–I_ interaction brings an electron transfer of about 0.27e from n_F_ orbital to σ*_N–I_ antibonding orbital. The higher occupancy of the σ*_N–I_ antibonding orbital indicates that the resonance structure H_3_N–I^+^:F^−^ is likely to be less stable than the resonance structure H_3_N: I–F for the NH_3_IF complex. Therefore, the H_3_N: I–F structure is predicted to be the best NLS, and it can form a strong resonance mixing with the H_3_N–I^+^:F^−^ structure.

Table 2 presents the calculated NRT weightings (*w*) for all studied BXY complexes, from which the best NLS (corresponding to the maximum *w*) can be identified directly. As expected, for the NH_3_IF complex the best NLS is indeed the resonance structure H_3_N: I–F, not H_3_N–I^+^:F^−^. Actually, the *w*_I_ of the B: X–Y structure is always greater than the *w*_II_ of the B–X^+^:Y^−^ structure for each of the studied BXY here, and the sum of *w*_I_ and *w*_II_ is always close to 100% (from 98.29% to 100.00%). What is more, when the Lewis base B is H_2_O in a halogen-bonded complex, the resonance structure B: X–Y is obviously dominant and the complex BXY does not belong to the 3c/4e ω-bonding family. However, when it turns to the NH_3_XY series, the *w*_II_ of the structure B–X^+^:Y^−^ are quite impressive for NH_3_ClF, NH_3_BrF, and NH_3_IF complexes. For instance, their *w*_II_ values are 22.81%, 28.80%, and even 32.27%, respectively. According to the proposal by Weinhold et al. [21], these three BXY complexes can be classified as the 3c/4e ω-bonding family. Each of them can be described as a resonance hybrid of B: X–Y ↔ B–X^+^:Y^−^ with strong mixing.

### 2.3. Charge-Shift Bonding in a Halogen-Bonded Complex

The AIM method is a powerful tool for studying covalent bonds, closed-shell interactions, charge-shift bonds, etc. Initially, Bader and Essen [47] proposed that covalent bonds can be identified by large total density *ρ*(r) and negative Laplacian ∇^2^*ρ*(r) at the BCP, while closed-shell interactions can be characterized by the combination of small *ρ*(r) and positive ∇^2^*ρ*(r). However, a special case is the combination of large *ρ*(r) and positive (or small) ∇^2^*ρ*(r), which is not originally addressed in the AIM theory. Shaik et al. [2,4,5] were the first to propose this combination as an important AIM indicator of homonuclear CS bonding, such as the O–O bond in H_2_O_2_, the F–F bond in F_2_, and the Cl–Cl bond in Cl_2_. In their recent publications [6,18], such an AIM indicator also was effective for the newly reported heteronuclear CS bonding, such as the N–B bond in H_3_N–BH_3_, the N–Cu bond in H_3_N–Cu^+^, and the F–NO_2_ bond in the selected explosive molecule. Therefore, in our recent study on the Cu/Ag/Au-bonded complexes [16], this AIM indicator was employed to illustrate their CS bonding characters as well.

**Figure 4:**
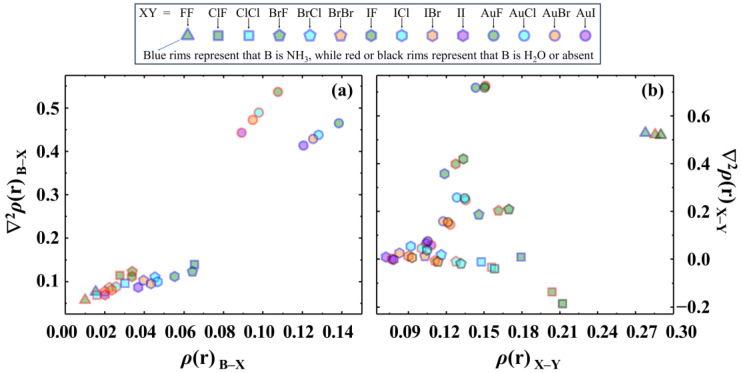
Calculated *ρ*(r) and ∇^2^*ρ*(r) at the BCPs of B–X (**a**) and X–Y (**b**) bonds in all studied species (in a.u.). The blue, red, and black rims represent that the Lewis base B is NH_3_, H_2_O, and absent (free XY), respectively. The symbols triangle, square, pentagon, hexagon, and circle represent that the atom X is F, Cl, Br, I, and Au, respectively. The green, cyan, orange, and purple centers represent that the atom Y is F, Cl, Br, and I, respectively.

The calculated total density *ρ*(r) and Laplacian ∇^2^*ρ*(r) at the BCPs of B–X and X–Y bonds in all studied BXY complexes are presented in Figure 4a,b, respectively, with the corresponding values listed in Table S8 in the SI. For comparison, the AIM descriptors of BAuY are also presented. It is evident that the *ρ*(r) is significant and the ∇^2^*ρ*(r) is positive at the BCPs of both B–Au and Au–Y bonds in the coinage-metal bonded BAuY. However, for the B–X bonds in the halogen-bonded complexes, the values of *ρ*(r) are mostly low and the ∇^2^*ρ*(r) are clearly positive (or small). Consequently, the majority of halogen bonds here conform to the AIM indicator of closed-shell interaction. However, there are still several BXY complexes, such as NH_3_ClF, NH_3_BrF, and NH_3_IF, whose halogen bonds are the CS bonding based on the relatively large *ρ*(r) values (more than 0.05 a.u.) and positive ∇^2^*ρ*(r) values.

In Figure 4b, we also present the calculated *ρ*(r) and ∇^2^*ρ*(r) values at the BCPs in free di-halogens, with corresponding values listed in the last two columns of Table S8. It is worth mentioning that Shaik et al. have previously reported that di-halogens (F_2_, ClF, Cl_2_, BrF, BrCl, Br_2_, etc.) [2,48] belong to the CS bonding family. As illustrated in Figure 4b, the combination of large *ρ*(r) and positive (or small) ∇^2^*ρ*(r) effectively probes the CS bonding in these free di-halogens, except for the ClF species, whose ∇^2^*ρ*(r) is −0.185 a.u. This particular case is like the HF molecule, in which the reported values of *ρ*(r) and ∇^2^*ρ*(r) at the BCP are 0.38 and −2.52, respectively [4]. However, other calculations have demonstrated that both ClF and HF are indeed typical CS bonding molecules. In any case, the bonding in ClF, BrF, and IF is CS bonding. The foregoing discussion is an effect to show the CS bonding nature of X–Y in free di-halogens, namely ClF, BrF, and IF. Now we begin a discussion of X–Y bonding in stronger halogen-bonded complexes. It can be seen in Table S8 and Figure 4b that both *ρ*(r) and ∇^2^*ρ*(r) at the BCP of the X–Y bond in NH_3_ClF, NH_3_BrF, and NH_3_IF meet the AIM criteria of CS bonds. Thus, the X–Y bond in NH_3_ClF, NH_3_BrF, and NH_3_IF is also CS bonding. Such results imply that the X–Y bond of these three complexes retains the free X–Y bonding nature upon complexation.

In addition to the combination of large *ρ*(r) and positive ∇^2^*ρ*(r), the delocalization index (DI) serves as an alternative AIM indicator for CS bonding. CS bonding implies significant delocalization arising from the lone pairs of the bonded atoms, leading to the DI values expected to exceed its formal bond order. For example, the DI values of the charge-shift bonds F–F (in F_2_) and B–N (in BH_3_NH_3_) are 1.24 and 0.38, respectively, in the report by Silvi et al. [48] Recently, Galland et al. [19,20] employed the DI to explain the CS bonding nature in At_3_C–At···Cl^−^ and C_6_At_6_ species as well. Table 3 presents the calculated DI values of B–X (DI_B–X_) and X–Y (DI_X–Y_) bonding interactions in all studied molecules. Notably, the DI_B–X_ value in halogen-bonded NH_3_XY is larger than that in the corresponding H_2_OXY, and it is even greater than 0.5 in NH_3_ClF, NH_3_BrF, and NH_3_IF. These results suggest that among all studied BXY complexes, the halogen bonds in these three complexes exhibit a relatively prominent CS bonding nature. In contrast, the DI_X–Y_ values are significant in all studied BXY, with almost all of them exceeding 1.0. Furthermore, the DI_X–Y_ value in each of the studied BXY complexes is smaller than that in the corresponding free XY di-halogen, especially in the series of NH_3_XY. Nevertheless, each of the studied X–Y bonds, whether it is in BXY or free XY, displays a clear CS bonding character.

In summary, based on the above AIM analyses, the studied BXY can be classified into two types. One has a weak halogen bond that exhibits a closed-shell interaction similar to the van der Waals forces, while the other displays the CS bonding nature with a stronger B–X interaction. In addition, all studied free XY di-halogens also belong to the CS bonding family. Consequently, we anticipate that the CS bonding nature established in all studied BXY complexes may be closely related to the free XY di-halogens.

### 2.4. CS Bonding Propensity in a Halogen-Bonded Complex

**Figure 5:**
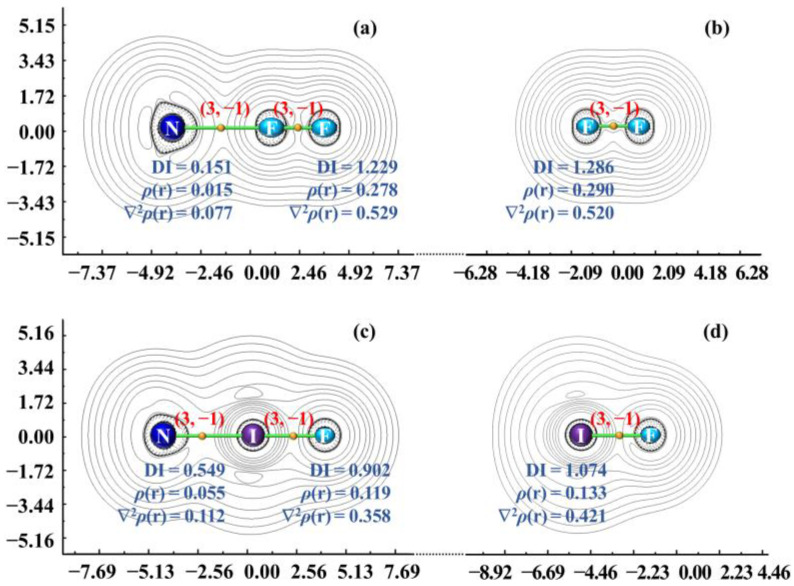
Two-dimensional representations of the Laplacian of the electron density for NH_3_FF (**a**), FF (**b**), NH_3_IF (**c**), and IF (**d**). Length unit: Bohr. All values at the BCPs are in a.u.

The foregoing analysis has revealed that the majority of the studied halogen bonds do not fall within the 3c/4e ω-bonding family in the NBO/NRT framework and that they do not exhibit the CS bonding nature based on the AIM descriptors. However, there are a few cases, such as the 3c/4e ω-bonded NH_3_ClF, NH_3_BrF, and NH_3_IF. Their halogen bonds do display the CS bonding characters. Additionally, it is essential to emphasize that the free di-halogens ClF, BrF, and IF also exhibit the CS bonding nature. It raises the following question: is there a connection of CS bonding between the halogen-bonded complexes BXY and the free di-halogens XY? However, a direct connection between them is not straightforward, because one refers to a two-center molecule and the other to a species with three more centers. To address this question, we present two representative examples in Figure 5. Now let us observe certain AIM characteristic aspects of the 3c/4e ω-bonded NH_3_IF complex. The AIM quantities indicate that the N–I and I–F bonds in NH_3_IF are the same in the CS bonding nature. The complex NH_3_IF clearly distinguishes its bonding from the NH_3_FF species. One of the reasons for this distinction is that the weak van der Waals forces between molecules are generally not considered as leading to chemical-bond formation. Similarly, for NH_3_ClF and NH_3_BrF complexes, we arrive at the same conclusion. While our discussion has thus far focused on the halogen CS bonding in studied BXY complexes, we continue to explore more examples, including the Cu/Ag/Au CS bonding. Indeed, as emphasized in our latest publication [16] concerning the Cu/Ag/Au bonding in BMY complexes, an intrinsic feature of B–M–Y is the appearance of the same CS bonding nature in B–M and M–Y bonds. Here, note that the coinage metal halides (free MY) also belong to the CS bonding family [8]. It becomes clear that the free MY tends to carry over its CS bonding nature to the corresponding BMY upon complexation.

All in all, for the studied 3c/4e ω-bonded BXY, a strong connection of the CS bonding nature exists between the B–X bond in BXY complexes and the X–Y bond in free XY. If the free XY has the CS bonding characters, the halogen bonding in 3c/4e ω-bonded BXY complexes trends to form CS bonding, just as the free MY shows its CS bonding propensity in BMY complexes.

## 3. Computational Details

The geometry optimization of each studied molecule here was carried out with the Gaussian 09 [49] program at the level of the second-order Møller–Plesset (MP2) [50] theory. The systematically convergent triple-ζ basis sets (aug-cc-pVTZ-PP) [51], which include small-core energy-consistent relativistic pseudopotentials (PP) to account for relativistic effects, were used for the heavy I and Au atoms, while the augmented correlation-consistent triple-ζ Dunning basis sets (aug-cc-pVTZ) [52] were adopted for the remaining atoms. The vibrational frequencies and dissociation energies of B–X and X–Y bonds in all studied molecules were calculated at the same level of theory and basis sets, and it is confirmed that the optimized geometrical structures correspond to the true minimum character because no imaginary frequencies were found in any case. In addition, the calculations of geometry optimization, vibrational frequency, and dissociation energy were also performed at the level of coupled-cluster with single and double excitations (CCSD) [53] theory in combination with def2-TZVPPD [54], which is a triple-ζ valence all-electron basis set augmented with two sets of polarization and diffuse basis functions. Notably, the homolytic bond cleavage enthalpies calculated at the CCSD level are more consistent with the higher CCSD(T) [55] level. Table S9 shows the comparisons of the B3LYP [56,57], MP2, CCSD, and CCSD(T) computational levels in combination with different basis sets for optimizing bond lengths of the free XY molecules. According to the relative mean deviations (*RMDs*), which were calculated with respect to the corresponding available experimental values, we find that the optimized bond lengths obtained at the CCSD(T) level in combination with aug-cc-pVQZ (-PP) [58,59,60] basis sets are the most accurate (*RMD* = 0.34%). Similarly, as shown in Table S10, the vibrational frequencies of free X–Y bonds calculated at the CCSD(T)/aug-cc-pVQZ (-PP) computational level are also the closest to the experimental values (*RMD* = 0.98%). Although using such a high computational level can obtain relatively accurate data, it is too expensive to deal with complex systems. We take the dissociation energies calculated at the CCSD(T)/aug-cc-pVQZ (-PP) computational level as the benchmark to test the accuracy at other different calculation levels in Table S11 and find that the results obtained at the CCSD/def2-TZVPPD computational level are accurate with an acceptable calculation cost. Note that the geometry optimizations at the MP2 level are generally closer to the experimental results than those at the CCSD level. Then, all subsequent NBO/NRT/AIM analyses are based on the optimized molecular structures at the MP2 level. The NBO/NRT [43,44,45,46] calculations were carried out with the NBOPro 6.0 [61,62] program to investigate the bonding of each optimized molecule, and the NBOView 2.0 module was employed to obtain the NBO orbital graphics. The electron density in the quantum theory of atoms in molecules [3] was conducted using the topology analysis module implemented in the Multiwfn 3.7 [63] program. Being consistent with our recent work [16], the delocalization index (DI) was also calculated at the B3LYP level.

## 4. Conclusions

This study investigated the CS bonding in halogen-bonded BXY complexes by using NBO/NRT/AIM methods. By analyzing the calculated equilibrium lengths, vibrational frequencies, and dissociation energies of the B–X and X–Y bonds, we observed that certain complexes, such as NH_3_ClF, NH_3_BrF, and NH_3_IF, exhibit relatively stronger halogen bonds. Through NBO/NRT analyses, each of the NH_3_ClF, NH_3_BrF, and NH_3_IF species can be described as a resonance hybrid B: X–Y ↔ B–X^+^:Y^−^ with strong mixing, unequivocally belonging to the 3c/4e ω-bonding family. The AIM analyses showed that the halogen bonds in these 3c/4e ω-bonded complexes exhibit CS bonding characters, whereas other halogen-bonded complexes can be described as closed-shell interaction van der Waals forces. Moreover, drawing upon further CS bonding examples from our recent study on Cu/Ag/Au-bonded BMY complexes, we found a novel CS bonding propensity. It reveals that the halogen/coinage-metal bonding in 3c/4e ω-bonded BXY/MXY tends to display the CS bonding characters if the free XY/MY also belongs to the CS bonding family. In essence, the CS bonding nature present in the free XY/MY molecule carries over the 3c/4e ω-bonded BXY/MXY.

Finally, we stress two requirements that the CS bonding propensity must meet. In addition to demanding that the free X–Y/M–Y is CS bonding, it usually also requires that the halogen/coinage-metal bonded BXY/BMY belongs to the ω-bonded species. Therefore, this CS bonding propensity is exclusively applicable to 3c/4e ω-bonded molecules.

We anticipate that this CS bonding propensity could potentially serve as a valuable tool for probing the CS bonding nature in numerous other 3c/4e ω-bonded molecules. However, we acknowledge that further related studies are currently underway to confirm whether this propensity can be considered a rule rather than an exception.

## Figures and Tables

**Table 1:** Calculated equilibrium bond lengths at the MP2/aug-cc-pVTZ (-PP) computational level.

	B	NH_3_		H_2_O		Free
XY		*r*_B–X_ (Å)	*r*_X–Y_ (Å)	*r*_B–X_ (Å)	*r*_X–Y_ (Å)	*r*_X–Y_ (Å)
FF		2.595 (2.71) [29]	1.416 (1.43) [30]	2.649 (2.70) [31]	1.407	1.401 (1.41) [30]
ClF		2.232 (2.37) [32]	1.714 (1.70) [30]	2.515 (2.54) [33]	1.656	1.638 (1.63) [30]
ClCl		2.592 (2.73) [34]	2.034 (2.00) [34]	2.771 (2.81) [35]	2.008 (2.01) [24]	1.999 (1.99) [30]
BrF		2.292 (2.34) [25]	1.829 (1.82) [25]	2.493	1.780	1.758 (1.76) [25]
BrCl		2.469 (2.63) [36]	2.203 (2.19) [36]	2.698 (2.74) [37]	2.155 (2.15) [24]	2.138 (2.14) [25]
BrBr		2.538 (2.72) [38]	2.332 (2.34) [38]	2.757 (2.80) [39]	2.292 (2.29) [24]	2.279 (2.28) [25]
IF		2.443	1.976	2.585	1.941	1.920
ICl		2.540 (2.54) [24]	2.390 (2.39) [24]	2.731 (2.78) [40]	2.343 (2.34) [24]	2.321 (2.32) [24]
IBr		2.583 (2.58) [24]	2.530 (2.53) [24]	2.781 (2.78) [24]	2.484 (2.48) [24]	2.465 (2.47) [24]
II		2.672 (2.67) [24]	2.717 (2.72) [24]	2.868 (2.87) [24]	2.678 (2.68) [24]	2.663 (2.66) [24]
AuF		1.995	1.909	2.038	1.893	1.902 (1.92) [41]
AuCl		2.029	2.199	2.079	2.178	2.174 (2.20) [41]
AuBr		2.040	2.310	2.094	2.289	2.284 (2.32) [41]
AuI		2.058	2.466	2.121	2.444	2.436 (2.47) [42]

The values in brackets are available experimental or calculated data. The values for the BAuY series from Ref. [16] are introduced to compare the halogen bonds with the coinage-metal bonds. The calculated comparisons from Ref. [24] at the MP2/aug-cc-pVTZ (-PP) level. The calculated comparisons from Ref. [25] at the CCSD(T)/aug-cc-pVTZ (-PP) level.

**Table 2:** NRT weightings *w*_I_ and *w*_II_ of B: X–Y and B–X^+^:Y^−^ for BXY.

	B	NH_3_		H_2_O	
XY		*w*_I_	*w*_II_	*w*_I_	*w*_II_
FF		98.88%	0.85%	99.85%	0.15%
ClF		75.74%	22.81%	94.48%	4.94%
ClCl		94.94%	3.99%	98.86%	0.88%
BrF		70.47%	28.80%	90.55%	8.67%
BrCl		87.69%	10.60%	97.23%	2.26%
BrBr		91.06%	7.26%	98.06%	1.51%
IF		67.37%	32.27%	86.53%	12.82%
ICl		82.30%	17.16%	94.74%	4.55%
IBr		85.87%	13.41%	96.16%	3.20%
II		90.57%	7.76%	97.67%	1.82%
AuF		49.59%	49.26%	65.68%	33.22%
AuCl		62.87%	36.27%	76.69%	22.00%
AuBr		66.17%	32.85%	79.05%	19.51%
AuI		69.96%	29.12%	82.29%	16.12%

The values for the BAuY series from Ref. [16] are introduced to compare the halogen bonds with the coinage-metal bonds.

**Table 3:** Calculated delocalization index of B–X (DI_B–X_) and X–Y (DI_X–Y_) bonding interactions in all studied molecules at the B3LYP/aug-cc-pVTZ (-PP) computational level.

	B	NH_3_		H_2_O		Free
XY		DI_N–X_	DI_X–Y_	DI_O–X_	DI_X–Y_	DI_X–Y_
FF		0.151	1.229	0.077	1.269	1.286
ClF		0.533	1.051	0.250	1.184	1.253
ClCl		0.283	1.340	0.144	1.414	1.446
BrF		0.563	0.976	0.316	1.087	1.175
BrCl		0.414	1.221	0.208	1.350	1.415
BrBr		0.368	1.265	0.186	1.371	1.419
IF		0.549	0.902	0.344	0.982	1.074
ICl		0.464	1.126	0.260	1.247	1.340
IBr		0.432	1.186	0.237	1.305	1.386
II		0.374	1.258	0.202	1.354	1.408
AuF		0.913	0.919	0.720	0.976	1.098
AuCl		0.849	1.244	0.652	1.318	1.464
AuBr		0.831	1.311	0.632	1.390	1.533
AuI		0.803	1.403	0.600	1.479	1.608

The values for the BAuY series from Ref. [16] are introduced to compare the halogen bonds with the coinage-metal bonds.

## Data Availability

The data presented in this study are available in the main text and the Supplementary Materials.

## Supplementary Materials

The following supporting information can be downloaded at: https://www.mdpi.com/article/10.3390/molecules28176212/s1, Figure S1: Correlation plots for bond length and bond frequency in NH_3_IY and NH_3_AuY series. Figure S2: NBO orbital contour diagrams for n_N_→σ*_I–F_ and n_F_→σ*_N–I_ in the NH_3_IF complex. Tables S1–S2: Calculated vibrational frequencies and dissociation energies of B–X and X–Y bonds in all studied molecules at the MP2/aug-cc-pVTZ (-PP) computational level. Tables S3–S5: Calculated equilibrium lengths, vibrational frequencies, and dissociation energies of B–X and X–Y bonds in all studied molecules at the CCSD/def2-TZVPPD computational level. Table S6: Calculated second-order perturbation stabilization energy Δ*E*^(2)^_D→A_ (kcal/mol) due to the donor-acceptor interactions n_B_→σ*_X–Y_ and n_Y_→σ*_B–X_. Table S7: Calculated orbital occupancies (e) of lone pair orbital n_B_ and antibonding orbital σ*_M–X_ arising from n_B_→σ*_M–X_, as well as those of n_X_ and σ*_B–M_ arising from n_X_→σ*_B–M_. Table S8: Calculated *ρ*(r) and ∇^2^*ρ*(r) at the BCPs of B–X and X–Y bonds in complex BXY and free dihalide XY. Tables S9–S11: Comparisons of the B3LYP, MP2, CCSD, and CCSD(T) computational levels in combination with different basis sets for calculating equilibrium lengths, vibrational frequencies, and dissociation energies of the free XY molecules. References [16,24,25,29,30,31,32,33,34,35,36,37,38,39,40,41,42] are cited in the supplementary materials.

## References

[1] Shaik S., Maitre P., Sini G., Hiberty P.C. (1992). The Charge-Shift Bonding Concept. Electron-Pair Bonds with Very Large Ionic-Covalent Resonance Energies. J. Am. Chem. Soc..

[2] Shaik S., Danovich D., Wu W., Hiberty P.C. (2009). Charge-Shift Bonding and Its Manifestations in Chemistry. Nat. Chem..

[3] Bader R.F.W. (1991). A quantum theory of molecular structure and its applications. Chem. Rev..

[4] Zhang L., Ying F., Wu W., Hiberty P.C., Shaik S. (2009). Topology of Electron Charge Density for Chemical Bonds from Valence Bond Theory: A Probe of Bonding Types. Chem.-A Eur. J..

[5] Shaik S., Danovich D., Braida B., Wu W., Hiberty P.C., Mingos D.M.P. (2016). New Landscape of Electron-Pair Bonding: Covalent, Ionic, and Charge-Shift Bonds. Chemical Bond II: 100 Years Old and Getting Stronger.

[6] Shaik S., Danovich D., Galbraith J.M., Braida B., Wu W., Hiberty P.C. (2020). Charge-Shift Bonding: A New and Unique Form of Bonding. Angew. Chem.-Int. Ed..

[7] Radenkovic S., Danovich D., Shaik S., Hiberty P.C., Braida B. (2017). The Nature of Bonding in Metal-Metal Singly Bonded Coinage Metal Dimers: Cu_2_, Ag_2_ and Au_2_. Comput. Theor. Chem..

[8] Dordevic S., Radenkovic S., Shaik S., Braida B. (2022). On the Nature of the Bonding in Coinage Metal Halides. Molecules.

[9] Shaik S., Danovich D., Silvi B., Lauvergnat D.L., Hiberty P.C. (2005). Charge-Shift Bonding-A Class of Electron-Pair Bonds That Emerges from Valence Bond Theory and Is Supported by the Electron Localization Function Approach. Chem.-A Eur. J..

[10] Levine D.S., Head-Gordon M. (2017). Energy Decomposition Analysis of Single Bonds within Kohn-Sham Density Functional Theory. Proc. Natl. Acad. Sci. USA.

[11] Joy J., Danovich D., Kaupp M., Shaik S. (2020). Covalent vs Charge-Shift Nature of the Metal-Metal Bond in Transition Metal Complexes: A Unified Understanding. J. Am. Chem. Soc..

[12] Braida B., Hiberty P.C. (2013). The Essential Role of Charge-Shift Bonding in Hypervalent Prototype XeF_2_. Nat. Chem..

[13] Braida B., Ribeyre T., Hiberty P.C. (2014). A Valence Bond Model for Electron-Rich Hypervalent Species: Application to SF_n_ (n = 1, 2, 4), PF_5_, and ClF_3_. Chem.-A Eur. J..

[14] Zhang G., Su Y., Zou X., Fu L., Song J., Chen D., Sun C. (2020). Charge-Shift Bonding in Xenon Hydrides: An NBO/NRT Investigation on HXeY∙∙∙HX (Y = Cl, Br, I.; X = OH, Cl, Br, I, CCH, CN) via H-Xe Blue-Shift Phenomena. Front. Chem..

[15] Legon A.C., Walker N.R. (2018). What’s in a Name? ‘Coinage-Metal’ Non-Covalent Bonds and Their Definition. Phys. Chem. Chem. Phys..

[16] Song J., Fu L., Li H., Su Y., Wang Q., Zhang G., Wang J. (2023). Equivalent and Complement of the omega-Bonding Model and Charge-Shift Bonding Model: A Natural Bond Orbital/Natural Resonance Theory/Atoms in Molecules Investigation via Cu/Ag/Au Bonding in BMX (B = H_2_O, H_2_S, NH_3_, and PH_3_; M = Cu, Ag, and Au; and X. = F, Cl, Br, and I). J. Phys. Chem. Lett..

[17] Gershoni-Poranne R., Chen P. (2017). The Carbon-Nitrogen Bonds in Ammonium Compounds Are Charge Shift Bonds. Chem.-A Eur. J..

[18] Joy J., Danovich D., Shaik S. (2021). Nature of the Trigger Linkage in Explosive Materials Is a Charge-Shift Bond. J. Org. Chem..

[19] Sarr S., Graton J., Montavon G., Pilme J., Galland N. (2020). On the Interplay between Charge-Shift Bonding and Halogen Bonding. ChemPhysChem.

[20] Sarr S., Pilme J., Montavon G., Le Questel J.-Y., Galland N. (2021). Astatine Facing Janus: Halogen Bonding vs. Charge-Shift Bonding. Molecules.

[21] Weinhold F., Clark R.L. (2005). Valency and Bonding: A Natural Bond Orbital Donor-Acceptor Perspective.

[22] Desiraju G.R., Ho P.S., Kloo L., Legon A.C., Marquardt R., Metrangolo P., Politzer P., Resnati G., Rissanen K. (2013). Definition of the Halogen Bond (IUPAC Recommendations 2013). Pure Appl. Chem..

[23] Legon A.C. (2014). A Reduced Radial Potential Energy Function for the Halogen Bond and the Hydrogen Bond in Complexes B∙∙∙XY and B∙∙∙HX, Where X and Y Are Halogen Atoms. Phys. Chem. Chem. Phys..

[24] Alkorta I., Legon A.C. (2019). Systematic Behaviour of Electron Redistribution on Formation of Halogen-Bonded Complexes BXY, as Determined via XY Halogen Nuclear Quadrupole Coupling Constants. Phys. Chem. Chem. Phys..

[25] Metrangolo P., Resnati G. (2008). Halogen Bonding: Fundamentals and Applications. Structure and Bonding.

[26] Legon A.C. (2021). An Assessment of Radial Potential Functions for the Halogen Bond: Pseudo-Diatomic Models for Axially Symmetric Complexes B∙∙∙ClF (B=N_2_, CO, PH_3_, HCN, and NH_3_). Chempluschem.

[27] Hobza P., Rezac J. (2016). Introduction: Noncovalent Interactions. Chem. Rev..

[28] Sachs L. (2012). Applied Statistics: A Handbook of Techniques.

[29] Bloemink H.I., Hinds K., Holloway J.H., Legon A.C. (1995). Characterisation of A Pre-Reactive Intermediate in Gas-Phase Mixtures of Fluorine and Ammonia: The Rotational Spectrum of the H_3_N∙∙∙F_2_ Complex. Chem. Phys. Lett..

[30] Karpfen A. (2000). Charge-Transfer Complexes Between NH_3_ and the Halogens F_2_, ClF, and Cl_2_: An ab initio Study on the Intermolecular Interaction. J. Phys. Chem. A.

[31] Cooke S.A., Cotti G., Holloway J.H., Legon A.C. (1997). Detection and Characterization of a Pre-Reactive Complex in a Mixture of Water and Fluorine: Rotational Spectrum of H_2_O∙∙∙F_2_. Angew. Chem.-Int. Ed..

[32] Bloemink H.I., Evans C.M., Holloway J.H., Legon A.C. (1996). Is the Gas-Phase Complex of Ammonia and Chlorine Monofluoride H_3_N∙∙∙ClF or [H_3_NCl]^+^ ∙∙∙F^−^? Evidence from Rotational Spectroscopy. Chem. Phys. Lett..

[33] Davey J.B., Legon A.C., Thumwood J.M.A. (2001). Interaction of Water and Dichlorine in the Gas Phase: An Investigation of H_2_O∙∙∙Cl_2_ by Rotational Spectroscopy and ab initio Calculations. J. Chem. Phys..

[34] Legon A.C., Lister D.G., Thorn J.C. (1994). Nonreactive Interaction of Ammonia and Molecular Chlorine-Rotational Spectrum of the Charge-Transfer Complex H_3_N∙∙∙Cl_2_. J. Chem. Soc.-Faraday Trans..

[35] Legon A.C., Thumwood J.M.A., Waclawik E.R. (2002). The Interaction of Water and Dibromine in the Gas Phase: An Investigation of the Complex H_2_O∙∙∙Br_2_ by Rotational Spectroscopy and ab initio Calculations. Chem.-A Eur. J..

[36] Bloemink H.I., Legon A.C., Thorn J. (1995). ‘Charge-Transfer’Complexes of Ammonia with Halogens. Nature of the Binding in H_3_N∙∙∙BrCl from its Rotational Spectrum. J. Chem. Soc.-Faraday Trans..

[37] Cooke S.A., Cotti G., Evans C.M., Holloway J.H., Legon A.C. (1996). The Pre-Reactive Complex H_2_O∙∙∙ClF Identified in Mixtures of Water Vapour and Chlorine Monofluoride by Rotational Spectroscopy. Chem. Commun..

[38] Bloemink H.I., Legon A.C. (1995). The Complex H_3_N∙∙∙Br_2_ Characterized in the Gas Phase by Rotational Spectroscopy. J. Chem. Phys..

[39] Davey J.B., Legon A.C. (2001). Rotational Spectroscopy of the Gas Phase Complex of Water and Bromine Monochloride in the Microwave Region: Geometry, Binding Strength and Charge Transfer. Phys. Chem. Chem. Phys..

[40] Davey J.B., Legon A.C., Waclawik E.R. (2000). An Investigation of the Gas-Phase Complex of Water and Iodine Monochloride by Microwave Spectroscopy: Geometry, Binding Strength and Electron Redistribution. Phys. Chem. Chem. Phys..

[41] Guichemerre M., Chambaud G., Stoll H. (2002). Electronic Structure and Spectroscopy of Monohalides of Metals of Group I-B. Chem. Phys..

[42] Reynard L.M., Evans C.J., Gerry M.C.L. (2001). The Pure Rotational Spectrum of AuI. J. Mol. Spectrosc..

[43] Weinhold F. (2012). Natural Bond Orbital Analysis: A Critical Overview of Relationships to Alternative Bonding Perspectives. J. Comput. Chem..

[44] Glendening E.D., Badenhoop J.K., Weinhold F. (1998). Natural Resonance Theory: III. Chemical Applications. J. Comput. Chem..

[45] Glendening E.D., Weinhold F. (1998). Natural Resonance Theory: I. General Formalism. J. Comput. Chem..

[46] Glendening E.D., Weinhold F. (1998). Natural Resonance Theory: II. Natural Bond Order and Valency. J. Comput. Chem..

[47] Bader R.F.W., Essen H. (1984). The Characterization of Atomic Interactions. J. Chem. Phys..

[48] Silvi B., Gillespie R.J., Gatti C., Reedijk J., Poeppelmeier K. (2013). Electron Density Analysis. Comprehensive Inorganic Chemistry II.

[49] Frisch M.J., Trucks G.W., Schlegel H.B., Scuseria G.E., Robb M.A., Cheeseman J.R., Scalmani G., Barone V., Mennucci B., Petersson G.A. (2009). Gaussian 09.

[50] Head-Gordon M., Pople J.A., Frisch M.J. (1988). MP2 Energy Evaluation by Direct Nethods. Chem. Phys. Lett..

[51] Feller D. (1996). The Role of Databases in Support of Computational Chemistry Calculations. J. Comput. Chem..

[52] Dunning T.H. (1989). Gaussian Basis Sets for Use in Correlated Molecular Calculations. I. The Atoms Boron Through Neon and Hydrogen. J. Chem. Phys..

[53] Purvis G.D., Bartlett R.J. (1982). A Full Coupled-Cluster Singles and Doubles Model: The Inclusion of Disconnected Triples. J. Chem. Phys..

[54] Rappoport D., Furche F. (2010). Property-Optimized Gaussian Basis Sets for Molecular Response Calculations. J. Chem. Phys..

[55] Raghavachari K., Trucks G.W., Pople J.A., Headgordon M.A. (1989). 5th-Order Perturbation Comparison of Electron Correlation Theories. Chem. Phys. Lett..

[56] Lee C.T., Yang W.T., Parr R.G. (1988). Development of the Colle-Salvetti Correlation-Energy Formula into a Functional of the Electron-Density. Phys. Rev. B.

[57] Becke A.D. (1993). Density-Functional Thermochemistry. III. The Role of Exact Exchange. J. Chem. Phys..

[58] Woon D.E., Dunning T.H. (1993). Gaussian-Basis Sets for Use in Correlated Molecular Calculations III. The Atoms Aluminum Through Argon. J. Chem. Phys..

[59] Peterson K.A. (2003). Systematically Convergent Basis Sets with Relativistic Pseudopotentials. I. Correlation Consistent Basis Sets for the Post-D Group 13–15 Elements. J. Chem. Phys..

[60] Peterson K.A., Figgen D., Goll E., Stoll H., Dolg M. (2003). Systematically Convergent Basis Sets with Relativistic Pseudopotentials. II. Small-Core Pseudopotentials and Correlation Consistent Basis Sets for the Post-D Group 16-18 Elements. J. Chem. Phys..

[61] Glendening E.D., Landis C.R., Weinhold F. (2013). NBO 6.0: Natural Bond Orbital Analysis Program. J. Comput. Chem..

[62] Weinhold F. (2013). NBOPro 6.0.

[63] Lu T., Chen F.W. (2012). Multiwfn: A Multifunctional Wavefunction Analyzer. J. Comput. Chem..

