# Supplementary material for: Charge-Shift Bonding Propensity in Halogen-Bonded BXY (B Is a Small Lewis Base H2O or NH3; X and Y Are Halogen Atoms) Complexes: An NBO/NRT/AIM Investigation
Source: Molecules. 2023 Aug 24;28(17):6212. doi: 10.3390/molecules28176212 (PMC10489069; doi:10.3390/molecules28176212)
Supplement: Supplementary file 1 [file molecules-28-06212-s001.zip › molecules-2551375-supplementary.pdf]

Supporting Information

Charge-Shift Bonding Propensity in Halogen Bonded BXY (B Is a Small Lewis Base H_2O or NH_3; X and Y Are Halogen Atoms) Complexes: An NBO/NRT/AIM Investigation

Junjie Song [1,2], Mengyang Wang [1], Xiaocheng Xu [2], Qinghao Shao [1], Ying Zhao [1], Guiqiu Zhang [1,*] and Nan Sun [1,*]

[1] Key Laboratory of Molecular and Nano Probes, Ministry of Education, College of Chemistry, Chemical Engineering and Materials Science, Shandong Normal University, Jinan 250014, China

[2] Key Laboratory of Organic Optoelectronics and Molecular Engineering of the Ministry of Education, Department of Chemistry, Tsinghua University, Beijing 100084, China

* Correspondence: gqzhang@sdnu.edu.cn (G.Z.); sunnan@sdnu.edu.cn (N.S.)

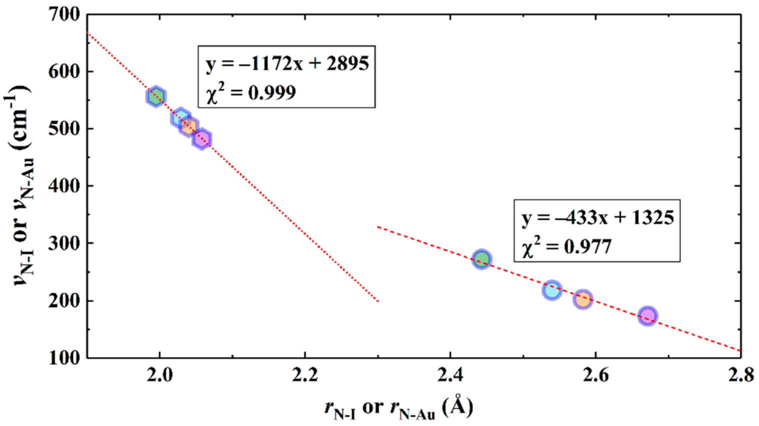

Figure S1. Correlation plots for bond length and bond frequency in NH_3IY and NH_3AuY series. The symbols hexagon and circle represent the I and Au atoms, respectively. The green, cyan, orange, and purple centers represent that the atom Y is F, Cl, Br, and I, respectively.

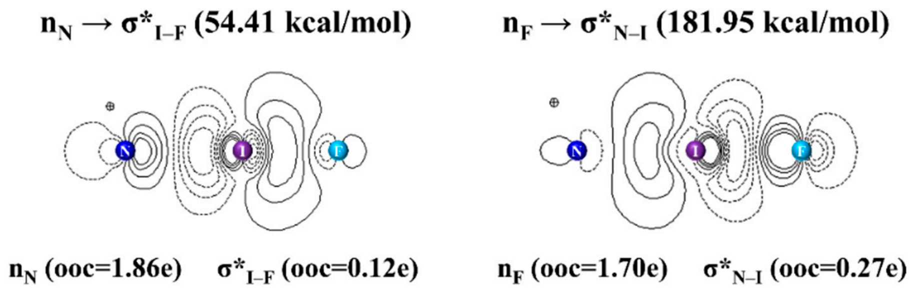

Figure S2. NBO orbital contour diagrams for $n_N \rightarrow \sigma^*_{I-F}$ and $n_F \rightarrow \sigma^*_{N-I}$ in NH_3IF complex.

Table S1. Calculated vibrational frequencies at the MP2/aug-cc-pVTZ (-PP) computational level.

XY \ B	NH3		H2O		Free
	$\nu_{N\text{–}X}$ (cm^{-1})	$\nu_{X\text{–}Y}$ (cm^{-1})	$\nu_{O\text{–}X}$ (cm^{-1})	$\nu_{X\text{–}Y}$ (cm^{-1})	$\nu_{X\text{–}Y}$ (cm^{-1})
FF	96	932	88	983	1004 (917) [1]
ClF	239	626	153	757	800 (786) [1]
ClCl	137	505	110	559	574 (560) [1]
BrF	267	576	178	653	693 (671) [2]
BrCl	187	393	134	443	461 (444) [2]
BrBr	168	294	122	331	341 (325) [2]
IF	272	558	187	604	634
ICl	218	353	150	388	406
IBr	202	250	139	275	286
II	173	205	125	225	230
AuF	556	622	466	638	584
AuCl	518	412	436	413	408
AuBr	504	285	411	292	287
AuI	482	229	382	236	236

The values in brackets are available experimental data. The values for BAuY series from Ref. [3] are introduced to compare the halogen bonds with the coinage-metal bonds.

Table S2. Calculated dissociation energies at the MP2/aug-cc-pVTZ (-PP) computational level.

XY \ B	NH3		H2O		Free
	$E_{N\text{–}X}$ (kcal/mol)	$E_{X\text{–}Y}$ (kcal/mol)	$E_{O\text{–}X}$ (kcal/mol)	$E_{X\text{–}Y}$ (kcal/mol)	$E_{X\text{–}Y}$ (kcal/mol)
FF	2.03	33.04	1.43	41.68	41.85
ClF	11.82	66.10	5.60	66.90	64.25
ClCl	5.48	53.50	3.16	58.20	57.99
BrF	16.78	74.73	8.37	72.33	67.58
BrCl	10.08	56.57	5.29	57.80	56.13
BrBr	8.60	52.37	4.63	54.41	53.40
IF	19.79	84.86	10.60	80.27	73.45
ICl	14.28	61.91	7.58	59.81	56.01
IBr	12.75	56.16	6.80	54.82	51.80
II	10.13	49.58	5.55	49.61	47.84
AuF	64.30	120.33	39.74	108.18	99.86
AuCl	56.23	109.74	33.71	99.63	99.01
AuBr	53.47	104.43	31.54	94.92	95.94
AuI	48.48	96.36	27.75	88.04	91.77

The values for BAuY series from Ref. [3] are introduced to compare the halogen bonds with the coinage-metal bonds.

Table S3. Calculated equilibrium bond lengths at the CCSD/def2-TZVPPD computational level.

B / XY	NH_3		H_2O		Free
	$r_{N–X}$ **(Å)**	$r_{X–Y}$ **(Å)**	$r_{O–X}$ **(Å)**	$r_{X–Y}$ **(Å)**	$r_{X–Y}$ **(Å)**
FF	2.821 (2.71) [4]	1.402 (1.43) [1]	2.795 (2.70) [5]	1.399	1.395 (1.41) [1]
ClF	2.402 (2.37) [6]	1.666 (1.70) [1]	2.631 (2.54) [7]	1.638	1.626 (1.63) [1]
ClCl	2.795 (2.73) [8]	2.015 (2.00) [8]	2.899 (2.81) [9]	2.004 (2.01) [10]	1.999 (1.99) [1]
BrF	2.400 (2.34) [2]	1.802 (1.71) [2]	2.612	1.768	1.754 (1.76) [2]
BrCl	2.656 (2.63) [11]	2.178 (2.19) [11]	2.829 (2.74) [12]	2.154 (2.15) [10]	2.144 (2.14) [2]
BrBr	2.735 (2.72) [13]	2.317 (2.34) [13]	2.894 (2.80) [14]	2.297 (2.29) [10]	2.289 (2.28) [2]
IF	2.512	1.949	2.673	1.920	1.902
ICl	2.652 (2.54) [10]	2.371 (2.39) [10]	2.829 (2.78) [15]	2.338 (2.34) [10]	2.322 (2.32) [10]
IBr	2.704 (2.58) [10]	2.519 (2.53) [10]	2.884 (2.78) [10]	2.487 (2.48) [10]	2.474 (2.47) [10]
II	2.829 (2.67) [10]	2.702 (2.72) [10]	2.987 (2.87) [10]	2.678 (2.68) [10]	2.668 (2.66) [10]
AuF	2.037	1.927	2.082	1.916	1.935 (1.92) [16]
AuCl	2.077	2.237	2.131	2.220	2.229 (2.20) [16]
AuBr	2.089	2.356	2.149	2.339	2.345 (2.32) [16]
AuI	2.108	2.513	2.181	2.493	2.496 (2.47) [17]

The values in brackets are available experimental or calculated data. The values for BAuY series from Ref. [3] are introduced to compare the halogen bonds with the coinage-metal bonds. The calculated comparisons from Ref. [10] at the MP2/aug-cc-pVTZ (-PP) level. The calculated comparisons from Ref. [2] at the CCSD(T)/aug-cc-pVTZ (-PP) level.

Table S4. Calculated vibrational frequencies of at the CCSD/def2-TZVPPD computational level.

B / XY	NH_3		H_2O		Free
	$\nu_{N–X}$ **(cm^{-1})**	$\nu_{X–Y}$ **(cm^{-1})**	$\nu_{O–X}$ **(cm^{-1})**	$\nu_{X–Y}$ **(cm^{-1})**	$\nu_{X–Y}$ **(cm^{-1})**
FF	79	982	74	997	1008 (917) [1]
ClF	177	709	131	785	808 (786) [1]
ClCl	112	535	97	559	565 (560) [1]
BrF	210	616	149	676	699 (671) [2]
BrCl	145	414	111	443	452 (444) [2]
BrBr	131	306	99	326	331 (325) [2]
IF	230	571	162	614	637
ICl	178	359	131	385	396
IBr	164	251	121	270	276
II	139	207	104	220	223
AuF	506	601	416	609	557
AuCl	469	383	404	364	375
AuBr	456	263	363	266	261
AuI	435	208	334	212	210

The values in brackets are available experimental data. The values for BAuY series from Ref. [3] are introduced to compare the halogen bonds with the coinage-metal bonds.

Table S5. Calculated dissociation energies at the CCSD/def2-TZVPPD computational level.

XY \ B	NH_3		H_2O		Free
	$E_{N–X}$ (kcal/mol)	$E_{X–Y}$ (kcal/mol)	$E_{O–X}$ (kcal/mol)	$E_{X–Y}$ (kcal/mol)	$E_{X–Y}$ (kcal/mol)
FF	1.45	19.93	1.10	27.43	28.27
ClF	8.29	53.44	4.44	54.85	52.89
ClCl	4.02	45.99	2.62	49.84	49.71
BrF	12.38	60.00	6.44	58.43	54.82
BrCl	7.07	47.27	4.09	48.66	47.41
BrBr	5.88	43.17	3.47	45.15	44.51
IF	15.51	69.75	8.37	65.93	60.56
ICl	10.82	51.61	6.02	50.13	47.10
IBr	9.46	45.95	5.32	45.12	42.79
II	7.15	39.73	4.20	40.10	38.89
AuF	53.59	104.86	32.04	92.03	99.86
AuCl	46.41	96.81	26.84	85.97	99.01
AuBr	43.68	91.02	24.77	80.83	95.94
AuI	39.42	82.59	21.67	73.57	91.77

The values for BAuY series from Ref. [3] are introduced to compare the halogen bonds with the coinage-metal bonds.

Table S6. Second-order perturbation stabilization energy $\Delta E^{(2)}_{D\rightarrow A}$ (kcal/mol) due to the donor-acceptor interactions $n_B \rightarrow \sigma^*_{X–Y}$ ($\Delta E^{(2)}_1$) or $n_Y \rightarrow \sigma^*_{B–X}$ ($\Delta E^{(2)}_2$) in all studied BXY complexes, calculated at the MP2/aug-cc-pVTZ (-PP) computational level.

XY \ B	NH_3		H_2O	
	$\Delta E^{(2)}_1$	$\Delta E^{(2)}_2$	$\Delta E^{(2)}_1$	$\Delta E^{(2)}_2$
FF	2.87	4741.37	0.74	2116.07
ClF	52.95	352.28	10.44	522.16
ClCl	13.92	2504.61	3.64	3721.83
BrF	62.17	260.92	17.98	360.82
BrCl	34.19	688.14	8.48	1183.52
BrBr	26.85	1226.02	6.76	3052.30
IF	54.41	181.95	21.61	233.05
ICl	42.31	343.05	13.58	511.91
IBr	37.24	446.94	11.36	749.35
II	27.34	850.88	8.13	2130.48
AuF	173.04	178.43	92.75	167.20
AuCl	179.30	323.77	93.01	301.19
AuBr	174.54	358.83	90.02	343.01
AuI	159.11	433.76	80.46	412.41

The values for BAuY series from Ref. [3] are introduced to compare the halogen bonds with the coinage-metal bonds.

Table S7. Orbital occupancies (e) of lone pair orbital n_B and antibonding orbital $\sigma^*_{X–Y}$ arising from the hyperconjugation $n_B \rightarrow \sigma^*_{X–Y}$, as well as those of n_Y and $\sigma^*_{B–X}$ arising from $n_Y \rightarrow \sigma^*_{B–X}$, calculated at the MP2/aug-cc-pVTZ (-PP) computational level.

B / XY	NH_3				H_2O			
	$\mathbf{n_N}$	$\sigma^*_{X–Y}$	$\mathbf{n_Y}$	$\sigma^*_{N–X}$	$\mathbf{n_O}$	$\sigma^*_{X–Y}$	$\mathbf{n_Y}$	$\sigma^*_{O–X}$
FF	1.99	0.01	1.04	0.93	2.00	0.00	1.03	0.88
ClF	1.85	0.13	1.52	0.42	1.97	0.02	1.44	0.50
ClCl	1.95	0.04	1.09	0.85	1.99	0.01	1.04	0.89
BrF	1.84	0.14	1.61	0.34	1.96	0.04	1.54	0.41
BrCl	1.90	0.09	1.27	0.67	1.98	0.02	1.18	0.77
BrBr	1.92	0.07	1.14	0.80	1.98	0.02	1.06	0.89
IF	1.86	0.12	1.70	0.27	1.95	0.04	1.65	0.31
ICl	1.88	0.10	1.43	0.52	1.97	0.03	1.35	0.60
IBr	1.89	0.10	1.33	0.62	1.97	0.03	1.24	0.72
II	1.91	0.08	1.17	0.78	1.98	0.02	1.08	0.88
AuF	1.80	0.19	1.79	0.20	1.88	0.11	1.78	0.21
AuCl	1.82	0.18	1.67	0.31	1.90	0.10	1.65	0.33
AuBr	1.82	0.17	1.64	0.34	1.90	0.09	1.62	0.36
AuI	1.83	0.16	1.59	0.39	1.91	0.09	1.56	0.42

The values for BAuY series from Ref. [3] are introduced to compare the halogen bonds with the coinage-metal bonds.

Table S8. Calculated total density $\rho(r)$ and Laplacian $\nabla^2\rho(r)$ at the BCPs of B–X and X–Y bonds at the MP2/aug-cc-pVTZ (-PP) computational level. All values are in a.u.

B / XY	NH_3				H_2O				Free	
	$\rho(\mathbf{r})_{N–X}$	$\nabla^2\rho(\mathbf{r})_{N–X}$	$\rho(\mathbf{r})_{X–Y}$	$\nabla^2\rho(\mathbf{r})_{X–Y}$	$\rho(\mathbf{r})_{O–X}$	$\nabla^2\rho(\mathbf{r})_{O–X}$	$\rho(\mathbf{r})_{X–Y}$	$\nabla^2\rho(\mathbf{r})_{X–Y}$	$\rho(\mathbf{r})_{X–Y}$	$\nabla^2\rho(\mathbf{r})_{X–Y}$
FF	0.015	0.077	0.278	0.529	0.010	0.058	0.285	0.522	0.290	0.520
ClF	0.065	0.140	0.179	0.010	0.027	0.115	0.204	−0.136	0.212	−0.185
ClCl	0.030	0.096	0.148	−0.010	0.016	0.069	0.156	−0.033	0.158	−0.039
BrF	0.064	0.123	0.146	0.187	0.034	0.123	0.161	0.204	0.170	0.209
BrCl	0.045	0.111	0.116	0.019	0.022	0.086	0.128	−0.009	0.132	−0.018
BrBr	0.040	0.103	0.103	0.013	0.020	0.077	0.111	−0.006	0.113	−0.010
IF	0.055	0.112	0.119	0.358	0.034	0.112	0.127	0.399	0.133	0.421
ICl	0.047	0.100	0.092	0.055	0.026	0.088	0.100	0.046	0.105	0.039
IBr	0.043	0.096	0.083	0.026	0.023	0.081	0.090	0.014	0.093	0.008
II	0.037	0.087	0.072	0.010	0.020	0.070	0.077	0.001	0.078	−0.001
AuF	0.138	0.465	0.143	0.719	0.107	0.537	0.151	0.727	0.150	0.719
AuCl	0.128	0.438	0.128	0.259	0.098	0.490	0.135	0.248	0.135	0.256
AuBr	0.125	0.429	0.117	0.160	0.095	0.473	0.123	0.146	0.121	0.156
AuI	0.120	0.414	0.104	0.067	0.089	0.443	0.108	0.059	0.105	0.076

The values for BAuY series from Ref. [3] are introduced to compare the halogen bonds with the coinage-metal bonds.

Table S9. Calculated equilibrium bond lengths (Å) for free XY species at the B3LYP, MP2, CCSD, and CCSD(T) levels in combination with different basis sets. The relative mean deviations (*RMDs*) were calculated with respect to the corresponding available experimental values (Exp.).

Free	aug-cc-pVTZ			def2-TZVPPD	aug-cc-pVQZ	Exp.
XY	B3LYP	MP2	CCSD(T)	CCSD	CCSD(T)	
FF	1.397	1.401	1.418	1.395	1.413	1.412 [1]
ClF	1.649	1.638	1.647	1.626	1.637	1.628 [1]
ClCl	2.024	1.999	2.020	1.999	2.003	1.988 [1]
BrF	1.775	1.758	1.765	1.754	1.756	1.759 [2]
BrCl	2.170	2.138	2.160	2.144	2.143	2.136 [2]
BrBr	2.316	2.279	2.302	2.289	2.283	2.281 [2]
IF	1.937	1.920	1.924	1.902	1.906	
ICl	2.357	2.321	2.342	2.322	2.326	
IBr	2.505	2.465	2.491	2.474	2.470	
II	2.703	2.663	2.692	2.668	2.664	
AuF	1.951	1.902	1.930	1.935	1.927	1.918 [16]
AuCl	2.248	2.174	2.220	2.229	2.212	2.199 [16]
AuBr	2.370	2.284	2.333	2.345	2.325	2.318 [16]
AuI	2.532	2.436	2.486	2.496	2.473	2.471 [17]
RMD	1.69%	0.70%	0.85%	0.72%	0.34%	

Table S10. Calculated vibrational frequencies (cm^{-1}) for free XY species at the B3LYP, MP2, CCSD, and CCSD(T) levels in combination with different basis sets. The relative mean deviations (*RMDs*) were calculated with respect to the corresponding available experimental values (Exp.).

Free	aug-cc-pVTZ			def2-TZVPPD	aug-cc-pVQZ	Exp.
XY	B3LYP	MP2	CCSD(T)	CCSD	CCSD(T)	
FF	1051	1004	916	1008	922	917 [1]
ClF	787	800	771	808	783	786 [1]
ClCl	537	574	541	565	553	560 [1]
BrF	675	693	673	699	679	671 [2]
BrCl	430	461	435	452	447	444 [2]
BrBr	316	341	322	331	331	325 [2]
IF	612	634	622	637	633	
ICl	383	406	385	396	392	
IBr	263	286	369	276	277	
II	213	230	216	223	224	
AuF	529	584	554	557	557	
AuCl	345	408	377	375	379	
AuBr	243	287	263	261	267	
AuI	196	236	215	210	217	
RMD	4.23%	4.28%	1.42%	3.59%	0.98%	

Table S11. Calculated dissociation energies (kcal/mol) for free XY species at the B3LYP, MP2, CCSD, and CCSD(T) levels in combination with different basis sets. The relative mean deviations (*RMDs*) were calculated with respect to the corresponding values at the CCSD(T)/aug-cc-pVQZ (-PP) level.

Free XY	aug-cc-pVTZ			def2-TZVPPD	aug-cc-pVQZ
	**B3LYP**	**MP2**	**CCSD(T)**	**CCSD**	**CCSD(T)**
FF	37.10	41.85	29.26	28.27	30.19
ClF	59.48	64.25	53.10	52.89	55.38
ClCl	54.02	57.99	49.26	49.71	52.10
BrF	61.99	67.58	56.55	54.82	58.46
BrCl	51.92	56.13	47.95	47.41	50.75
BrBr	48.90	53.40	45.83	44.51	48.46
IF	67.47	73.45	62.55	60.56	65.07
ICl	51.74	56.01	48.02	47.10	50.84
IBr	47.11	51.80	44.38	42.79	47.18
II	43.06	47.84	40.49	38.89	43.83
AuF	67.05	110.32	100.27	99.86	102.29
AuCl	62.49	107.80	98.81	99.01	101.80
AuBr	58.71	105.25	96.55	95.94	99.77
AuI	54.22	102.17	92.86	91.77	96.30
RMD	14.89%	12.12%	4.40%	6.06%	

References

1. Karpfen, A., Charge-Transfer Complexes Between NH_3 and the Halogens F_2, ClF, and Cl_2: An *ab initio* Study on the Intermolecular Interaction. *Journal of Physical Chemistry A* **2000,** *104* (29), 6871-6879.

2. Metrangolo, P.; Resnati, G., Halogen Bonding: Fundamentals and Applications. In *Structure and Bonding*, 2008; Vol. 126, pp 1-221.

3. Song, J.; Fu, L.; Li, H.; Su, Y.; Wang, Q.; Zhang, G.; Wang, J., Equivalent and Complement of the Omega-Bonding Model and Charge-Shift Bonding Model: A Natural Bond Orbital/Natural Resonance Theory/Atoms in Molecules Investigation via Cu/Ag/Au Bonding in BMX (B = H_2O, H_2S, NH_3, and PH_3; M = Cu, Ag, and Au; and X = F, Cl, Br, and I). *Journal of Physical Chemistry Letters* **2023,** *14* (23), 5226-5233.

4. Bloemink, H. I.; Hinds, K.; Holloway, J. H.; Legon, A. C., Characterisation of A Pre-Reactive Intermediate in Gas-Phase Mixtures of Fluorine and Ammonia: The Rotational Spectrum of the $H_3N\cdots F_2$ Complex. *Chemical Physics Letters* **1995,** *245* (6), 598-604.

5. Cooke, S. A.; Cotti, G.; Holloway, J. H.; Legon, A. C., Detection and Characterization of A Pre-Reactive Complex in A Mixture of Water and Fluorine: Rotational Spectrum of $H_2O\cdots F_2$. *Angewandte Chemie-International Edition* **1997,** *36* (1-2), 129-130.

6. Bloemink, H. I.; Evans, C. M.; Holloway, J. H.; Legon, A. C., Is the Gas-Phase Complex of Ammonia and Chlorine Monofluoride $H_3N\cdots ClF$ or $[H3NCl]^+\cdots F^-$? Evidence from Rotational Spectroscopy. *Chemical Physics Letters* **1996,** *248* (3), 260-268.

7. Davey, J. B.; Legon, A. C.; Thumwood, J. M. A., Interaction of Water and Dichlorine in the Gas Phase: An Investigation of $H_2O\cdots Cl_2$ by Rotational Spectroscopy and *ab initio* Calculations. *Journal of Chemical Physics* **2001,** *114* (14), 6190-6202.

8. Legon, A. C.; Lister, D. G.; Thorn, J. C., Nonreactive Interaction of Ammonia and Molecular Chlorine - Rotational Spectrum of the Charge-Transfer Complex $H_3N\cdots Cl_2$. *Journal of the Chemical Society-Faraday Transactions* **1994,** *90* (21), 3205-3212.

9. Legon, A. C.; Thumwood, J. M. A.; Waclawik, E. R., The Interaction of Water and Dibromine in the Gas Phase: An Investigation of the Complex $H_2O\cdots Br_2$ by Rotational Spectroscopy and *ab initio* Calculations. *Chemistry-a European Journal* **2002,** *8* (4), 940-950.

10. Alkorta, I.; Legon, A. C., Systematic Behaviour of Electron Redistribution on Formation of Halogen-Bonded Complexes BXY, as Determined via XY Halogen Nuclear Quadrupole Coupling Constants. *Physical Chemistry Chemical Physics* **2019,** *21* (31), 16914-16922.

11. Bloemink, H. I.; Legon, A. C.; Thorn, J., 'Charge-Transfer' Complexes of Ammonia with Halogens. Nature of the Binding in $H_3N\cdots BrCl$ from Its Rotational Spectrum. *Journal of the Chemical Society-Faraday Transactions* **1995,** *91* (5), 781-787.

12. Cooke, S. A.; Cotti, G.; Evans, C. M.; Holloway, J. H.; Legon, A. C., The Pre-Reactive Complex $H_2O\cdots ClF$ Identified in Mixtures of Water Vapour and Chlorine Monofluoride by Rotational Spectroscopy. *Chemical Communications* **1996,** (20), 2327-2328.

13. Bloemink, H. I.; Legon, A. C., The Complex $H_3N\cdots Br_2$ Characterized in the Gas Phase by Rotational Spectroscopy. *Journal of Chemical Physics* **1995,** *103* (3), 876-882.

14. Davey, J. B.; Legon, A. C., Rotational Spectroscopy of the Gas Phase Complex of Water and Bromine Monochloride in the Microwave Region: Geometry, Binding Strength and Charge Transfer. *Physical Chemistry Chemical Physics* **2001,** *3* (15), 3006-3011.

15. Davey, J. B.; Legon, A. C.; Waclawik, E. R., An Investigation of the Gas-Phase Complex of Water and Iodine Monochloride by Microwave Spectroscopy: Geometry, Binding Strength and Electron Redistribution. *Physical Chemistry Chemical Physics* **2000,** *2* (8), 1659-1665.

16. Guichemerre, M.; Chambaud, G.; Stoll, H., Electronic Structure and Spectroscopy of Monohalides of Metals of Group I-B. *Chemical Physics* **2002,** *280* (1), 71-102.

17. Reynard, L. M.; Evans, C. J.; Gerry, M. C. L., The Pure Rotational Spectrum of AuI. *Journal of Molecular Spectroscopy* **2001,** *205* (2), 344-346.
